# Pediatric traumatic brain injuries in war zones: a systematic literature review

**DOI:** 10.3389/fneur.2023.1253515

**Published:** 2023-09-06

**Authors:** Alex S. Aguirre, Kenny Rojas, Alcy R. Torres

**Affiliations:** Division of Pediatric Neurology, Department of Pediatrics, Boston Medical Center, Boston University Chobanian & Avedisian School of Medicine, Boston, MA, United States

**Keywords:** war, children, conflict, brain, injury

## Abstract

**Background:**

Pediatric casualties in war zones are a devastating consequence of armed conflicts, causing significant challenges for affected children, especially in the context of poor access to care. This study aimed to understand traumatic brain injuries (TBIs) in this high-risk population and to identify and provide information for the stakeholders, as well as to recognize severe long-term consequences and develop strategies to prevent them, thus minimizing their burden while aiding in the management of these cases.

**Methods:**

We carried out a systematic literature review following PRISMA guidelines to identify publications discussing traumatic brain injuries in children in the context of war zones, and we analyzed all the collected data.

**Results:**

Our study showed that head injuries were the most common casualty in war zones; male and female children were affected, and the mean age was 8–10 years. Most children were reported to be from Afghanistan, and blasts were the most common mechanism of injury. The mortality fluctuated from 3 to 47%.

**Conclusion:**

There is a lack of evidence-based information regarding the characterization, approach, and management of children with TBI in conflict zones. While the world finds ways to live in peace, there is an urgency to research, train, and deploy enough specialists to these areas, if governments are serious about improving outcomes for this population.

## 1. Introduction

Pediatric casualties in war zones are a devastating consequence of armed conflicts, causing significant physical, emotional, and cognitive challenges for affected children (1). War zones expose children to various forms of violence, such as being caught in crossfire or becoming victims of deliberate attacks, leading to head injuries that can result in severe head traumas such as traumatic brain injury (TBI). The consequences of pediatric TBIs in war zones are extensive, impacting not only the individual child but also their families and the community at large (2, 3).

War amplifies injuries due to the use of heavy artillery, explosive devices, and other weapons that cause substantial damage. The developing brains of children are particularly vulnerable, making them more susceptible to long-term cognitive and neurological impairments. The immediate effects of pediatric TBI in war zones can be life-threatening. Children may suffer from loss of consciousness, seizures, and respiratory difficulties, requiring urgent medical attention (4, 5).

Access to quality healthcare is often limited in war zones due to medical infrastructure destruction, lack of resources, and restricted movement. These challenges further exacerbate the difficulties in providing timely and appropriate care for children with TBIs. However, even when children with TBI receive initial medical care, the long-term consequences can be profound. Cognitive impairments, including memory deficits, attention problems, and executive functioning difficulties, are common. Emotional and behavioral changes, such as depression, anxiety, aggression, and impulsivity, may also arise due to the injury (1–3, 6).

These impairments can significantly affect a child's ability to learn, concentrate, and perform daily activities and their integration into society, leading to economic and public health burdens. Therefore, it is crucial that appropriate resources and research efforts are focused on comprehending the underlying mechanisms of TBIs in children in war zones. This understanding is essential for making accommodations considering the limited availability of specialized medical personnel, a shortage of rehabilitation facilities, and socioeconomic barriers that hinder comprehensive care (7). Our study aimed to clarify this topic, promoting awareness and prompting actions on this matter.

## 2. Methods

**Figure 1:**
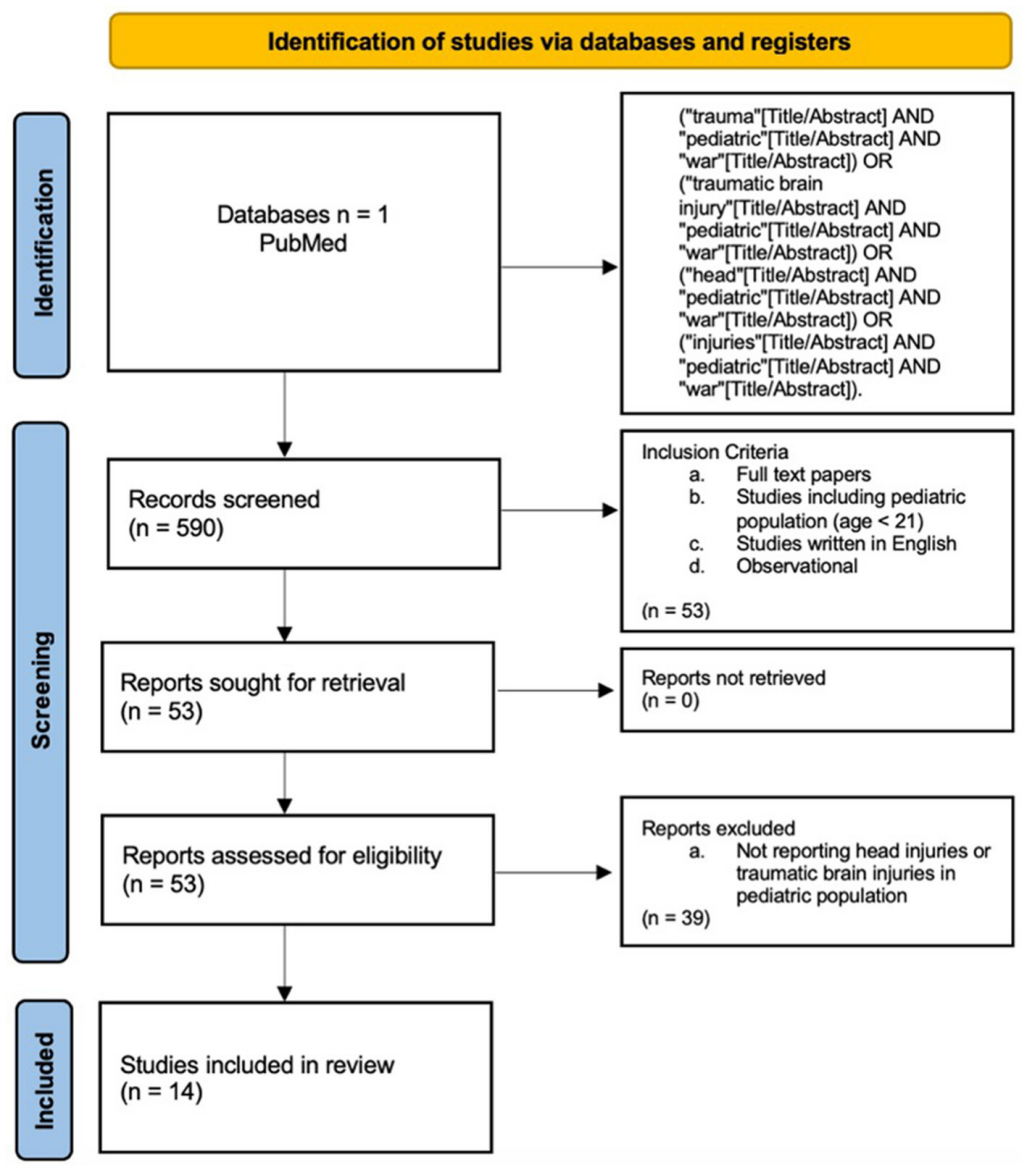
PRISMA protocol for this study. Identification, screening, and inclusion process.

We carried out a systematic review using the PRISMA protocol. Figure 1 shows the results of the study using this protocol.

### 2.1. Eligibility criteria and study selection

We selected studies written in English. Animal studies and articles that did not fulfill the aims of our study were excluded. We only included studies about trauma in war zones that contain an analysis of traumatic brain injury and head injuries in pediatric patients. We gathered outcomes of the mechanism of injury, the characteristics of the trauma, and the main conclusions.

### 2.2. Database and search strategy

We used the PubMed database for this systematic literature review. The search was conducted between 10 March and 16 June 2023. We used an advanced search strategy with the following terms: (“trauma”[Title/Abstract] AND “pediatric”[Title/Abstract] AND “war”[Title/Abstract]) OR (“traumatic brain injury”[Title/Abstract] AND “pediatric”[Title/Abstract] AND “war”[Title/Abstract]) OR (“head”[Title/Abstract] AND “pediatric”[Title/Abstract] AND “war”[Title/Abstract]) OR (“injuries”[Title/Abstract] AND “pediatric”[Title/Abstract] AND “war”[Title/Abstract]).

### 2.3. Data extraction and analysis

We collected the following information from each article: the author/year, methods, number of participants, and study design. We also extracted the main results, including each study's outcome measures and main limitations. We analyzed the studies' primary and secondary goals and gathered the main conclusions from each study.

### 2.4. Quality assessment

We used the Newcastle–Ottawa tool to perform the quality assessment in observational studies.

## 3. Study characteristics

We found 14 clinical trials that specifically discussed pediatric traumatic brain injuries in war zones, along with other pediatric casualties in this context.

Table 1 presents the demographics of each study. Our findings show that 50% (7/14) of the studies report pediatric TBIs that occurred in Afghanistan. The total sum of all pediatric samples found in these studies is 14,624. The mean age falls between 8 and 10 years. The most prevalent mechanism of injury is blast related to explosive devices (57%), followed by ballistic injuries caused by gunshots (29%) and fragment injuries (14%). TBI accounts for one of the most commonly reported injuries in war zones.

**Table 1:** Population demographics and most common mechanism of the TBI.

**References**	**Location**	**Pediatric sample**	**Mean age**	**Most common mechanism of injury**	**TBI**
Arul et al. (8)	Afghanistan	82	8	Blast	24/196 injuries (12%)
Cuenca et al. (9)	Iran and Afghanistan	495	8	Blast	478/495 (96%)
Cingoz et al. (10)	Syria	62	11.4 ± 6.3	Fragments	62/62 (100%)
Edwards et al. (11)	Iraq and Afghanistan	1,822	-	Blast	4,897/4,983 (98%) total Not specified for pediatric population
Er et al. (12)	Turkey	285	-	Blast	156/285 (54%)
Karakus et al. (13)	Syria	86	-	Ballistic	5/86 (6%)
Klimo et al. (14)	Iraq and Afghanistan	647	8	Blast	647/647 (100%)
Kocamer et al. (15)	Southern Boarder of Turkey	84	11.7+- 3.41	Ballistic	373/707 (52.7%) total Not specified for pediatric population
Maitland et al. (16)	Afghanistan	190	-	Blast	63/190 (33%)
McGuigan et al. (24)	Iraq	99	10.6	Ballistic	26/99 (26%)
McIntyre (25)	Syria	398	-	Ballistic	719/2,701 (26.6%) total Not specified for pediatric population
Naaman et al. (17)	Syria	117	12	Fragments	65/117 (56%)
Thompson et al. (8)	Afghanistan	295	12 Male, 8 Female	Blast	72/295 (24%)
Tovar et al. (5)	Afghanistan, Pakistan, Iraq, Somalia, Syria, and Yemen	9962	10.3	Blast	7,860/19,876 injuries (39.5%)

Description of the location, patients, mechanism of injury, and TBIs reported.

Table 2 shows the outcomes and conclusions of the studies included in this systematic review. Our findings show that 64% (9/14) of the studies used the New Injury Severity Score or the Injury Severity Score (NISS or ISS) to report injuries. The range of mortality lies between 3.4 and 47%. Cuenca et al. report that most TBIs generate an unspecific pattern but suggest that neurosurgeons should be prepared for intracranial hemorrhages (18). Naaman et al. support this idea (19). On a different note, Cingoz et al. report that intraparenchymal bone fragments caused most TBIs in their study and suggest that surgical treatment should be established in all patients using a Glasgow Coma Scale (GCS) higher than 3 and a radiological indication (20). Finally, Klimo et al. and Maitland et al. emphasize that male patients die less than female patients, possibly due to the inequality of healthcare access for female patients in these regions (17, 21).

**Table 2:** Characteristics and outcomes.

**References**	**Scale**	**Mortality**	**TBI characteristics**	**Other**
Arul et al. (8)	NISS	9%	Not reported	-
Cuenca et al. (9)	ISS	21%	14% of all pediatric war casualties are intracranial hemorrhage. Most are unspecified, mixed, or subdural	Authors suggest that military neurosurgeons should have the appropriate equipment and receive training for blast injuries causing intracranial hemorrhages
Cingoz et al. (10)	GCS/GOS	16.7–27.7%	Intraparenchymal bone fragments (51.6%), compression fractures (45.2%), intracerebral hematoma (27.4%), subdural hematoma (12.9%), and epidural hematoma (8.1%)	Authors suggest that surgical treatment should be established immediately in all patients with radiological indications and a GCS score higher than 3
Edwards et al. (11)	AIS/ISS	7.8%	28% of the injuries were to the head/neck. Head and cervical spine injuries are strongly associated with death	The authors suggest that a multidisciplinary team should provide care in these cases. Especially in all patients with an ISS higher than 15 and in those requiring a transfusion, for whom mortality is higher.
Er et al. (12)	ISS	9.3% estimated ED mortality, Brain injuries related death causes 72% (6/7)	The most common injured body part was the head. Linear fracture (24.5%) and contusion (17.3%) were the most common brain injuries	-
Karakus et al. (13)	-	4.65% total (not specified for pediatric population)	Not reported	Costs per case 15-69556 TL
Klimo et al. (14)	GCS	24.4%	Penetrating injuries were the most common. Isolated head injury was the main cause of death (24.1%)	Males were less likely to die as a result of an isolated head injury in Afghanistan
Kocamer et al. (15)	ASA/NISS	47%	Not reported	The highest mortality rates were observed in neurosurgical cases
Maitland et al. (16)	-	-	Pediatric blast casualties suffered significantly more head (36.9%) trauma compared to adults	Female trauma patients did not receive the same care as male patients, suggesting an inequality of access to trauma care for females within the native population
McGuigan et al. (24)	ISS	9%	High reports of penetrating cranial trauma (13%). Gunshot wounds were six times to die than victims of car crash	The authors emphasize that blast injuries are complex because they cause burns and inhalation injuries, blunt and penetrating wounds
McIntyre (25)	-	8.6% total (not specified for pediatric population)	Head injuries were the most reported injury (26.6%). One in six injuries were to children < 18 years old	-
Naaman et al. (17)	AIS/ISS, GCS, and PTS	3.4–6.7%	Head injuries were the most common affected injury (56%). The most common injuries were open skull fracture (21.4%), intracranial hemorrhage, and subdural hematoma. The most common cause of death was head injury	The authors found an association between head injury and injury severity. High AIS, ISS ≥ 25, and PTS < 6 are associated with head injuries.
Thompson et al. (8)	AIS/NISS	18.5%	Head injuries accounted for 88% of fatalities. Children between 2 and 7 years suffered more severe head or neck injuries (24.3%) compared with children aged between 8 and 15 years (19.8%). Thirty-four percent of head injuries were rated unsurvivable	-
Tovar et al. (5)	AIS	15.4%	Between terrorism-related and terrorism-unrelated, there is similar incidence of craniocerebral trauma (39.5 versus 43.1%)	Mortality rate of terrorism-related blasts is higher than terrorism-unrelated blasts (15.4 vs. 5.2%)

Description of the scales used to describe the TBIs, along with their characteristics. Mortality reports and other commentaries from the studies.

AIS, Abbreviated Injury Scale; ISS, Injury Severity Score; NISS, New Injury Severity Score; GCS, Glasgow Coma Scale; GOS, Glasgow Outcome Scale; TL, Turkish Lira; ED, Emergency Department; PTS, Pediatric Trauma Score.

Table 3 shows the quality assessment of the studies included in this systematic review.

**Table 3:** Quality assessment.

**References**	**Selection**	**Comparability**	**Exposure**	**Overall**
Arul et al. (22)	3	2	3	8
Cuenca et al. (18)	4	2	3	9
Cingoz et al. (20)	4	2	3	9
Edwards et al. (10)	2	2	3	7
Er et al. (14)	4	2	3	9
Karakus et al. (16)	3	2	3	8
Klimo et al. (17)	2	2	3	7
Kocamer et al. (23)	4	2	2	8
Maitland et al. (21)	4	2	3	9
McGuigan et al. (24)	3	2	2	7
McIntyre (25)	4	2	3	9
Naaman et al. (19)	2	2	3	7
Thompson et al. (8)	4	2	3	9
Tovar et al. (5)	3	2	3	9

A star system was used to perform quality assessments following the Newcastle–Ottawa Scale (NOS). A study was awarded one star for each numbered item within the selection and exposure categories. A maximum of two stars were awarded for comparability, three for exposure, and four for selection. The NOS ranges from zero to nine stars. We considered high-quality studies as those that achieved seven or more stars, medium-quality studies as those with four to six stars, and poor-quality studies as those with fewer than four stars.

## 4. Discussion

Multiple studies have demonstrated the vast consequences of war on children, from physical trauma to mental health problems (1, 6, 9, 11). Our study shows that TBIs in war zones have many mechanisms of injury that vary depending on the location and historical time. It is difficult to detail the characteristics of TBIs in every study, given that the scale used to represent the trauma is inconsistent and varies depending on the study. Additionally, most studies include “head trauma” or “head injury” as part of their TBI reports, even when the injuries range from minor bruises to injuries requiring neurosurgical consultation, making a detailed analysis of the TBIs difficult (5).

Our study focuses on all characteristics of TBIs; some data are provided by articles that failed to distinguish between pediatric and adult patient injuries but are good for understanding the background and characterization of TBIs in war. The most prevalent mechanism of injury is blast. Blast injuries usually cause more damage to the body than an isolated brain injury, which directly worsens the prognosis of the patients, increases the mortality of these cases, and raises concerns for bias in the collected data by making the reports unspecific information.

Blast injuries are linked directly to neurological effects due to the physical science behind them. Technically, explosive devices create shock waves with high-speed pressure changes that travel faster than sound. However, war explosions are more chaotic because they may involve multiple shock waves and reflections from surfaces. This causes various injuries from direct pressure effects on organs, flying objects that get thrown and hit people, and thermal effects. Electromagnetic disturbances may also be present, but their physiological impact is unclear (12).

Researchers are pivotal in advancing pediatric TBI understanding and treatment in war zones. Studies examining TBI's long-term outcomes, rehabilitation strategies, and psychosocial impact can guide evidence-based interventions.

### 4.1. Outcomes and mortality

Our study shows a range of mortality between 3.4 and 47%. However, each study's mean age and number of pediatric patients vary significantly. As mentioned earlier, the data are biased, causing marked variability. Nevertheless, it is evident that pediatric mortality is higher in war zones, especially when the children have a TBI associated with other lesions or when they have a TBI caused by war alone (17, 19–21). Pediatric population is more susceptible to dying from a TBI in war than an adult.

Most authors use the Abbreviated Injury Scale (AIS) or the ISS scale to report TBIs, while only three studies present GCS reports. Data analysis of these scales shows they are good for interpreting mortality and outcomes in patients with TBIs. While the AIS/ISS reports data concerning the physical and anatomical aspects of TBIs, GCS presents data from the functional and physiological aspects. There are no significant differences in the mortality reported from one scale to another, but it would be ideal for further studies to report on both anatomical and physiological scales (21, 23).

Some studies mentioned the difference between male and female children. However, this was not mentioned in light of any anatomical or physiological difference but in the context of culture. Some authors report that male injuries tend to be more commonly reported because of the geographical location of most wars (i.e., the middle east), where a male individual's life is more valuable than a female individual's (21). However, those reports may also be influenced by the historical role of men as house protectors; then, they would be more likely to be exposed to TBIs.

The only study that analyzed the treatment costs per case was conducted by Karakus et al., which noted that the value could be as high as 69,556 Turkish Liras, equivalent to 6.1 minimum wages per month in Turkey (16). Other studies have also analyzed the costs and financial burden of non-fatal TBIs in the United States, which in 2016 were 40.6 billion US dollars (26).

### 4.2. Mental health and physical consequences

War conflicts profoundly impact children's mental health, both during and after the conflicts. War experiences can leave deep psychological scars on young minds, often leading to long-lasting emotional, behavioral, and developmental issues. Understanding and addressing the mental health needs of children affected by war are crucial for their wellbeing and future prospects.

During war conflicts, children are exposed to a range of traumatic events such as violence, displacement, loss of loved ones, destruction of homes, and separation from their families. These experiences can lead to acute stress reactions, including fear, anxiety, depression, and post-traumatic stress disorders (PTSDs). Witnessing or experiencing violence can disrupt a child's normal brain development and have lifelong consequences.

Children in war-affected areas often lack access to essential resources and services, including mental healthcare. The destruction of healthcare infrastructure, limited availability of trained professionals, and societal stigma surrounding mental health exacerbate the challenges faced by these children. As a result, many suffer in silence, without the support they need to recover and rebuild their lives.

Even after the conflict ends, the effects of war on children's mental health can persist. They may continue to experience symptoms of trauma, depression, anxiety, and other mental health disorders. Additionally, the loss of education, disrupted social networks, and economic instability can further hinder their psychological wellbeing.

Additionally, children with TBIs suffer from severe physical consequences. These injuries can range from immediate life-threatening conditions to long-term impairments. Common physical consequences include motor impairments, sensory deficits, speech and language difficulties, cognitive impairments, and an increased risk of seizures and epilepsy (11, 13).

All these factors create an environment to develop chaos by increasing the pediatric risk of abuse and neglect due to limited access to basic needs and care. This is closely linked to more direct effects, such as toxic environmental exposures and the increased incidence of infectious diseases (13, 15).

### 4.3. Future recommendations

Addressing pediatric TBIs in war zones requires a multidisciplinary team. Efforts to solve the problem should focus on reducing armed conflicts and protecting children's rights during times of war. International humanitarian organizations and governments must work together to implement policies and interventions prioritizing children's safety and wellbeing, including strict regulations on using explosive weapons in populated areas.

Furthermore, healthcare infrastructure is critical in war-torn regions. Building and equipping medical facilities, training healthcare professionals in TBI management, and ensuring essential medical supplies are crucial steps. International aid organizations and governments should collaborate to provide financial support and technical expertise to strengthen healthcare systems. This, of course, should include a specific focus on pediatric TBI care.

Finally, rehabilitation services must also be expanded and tailored to meet the unique needs of children with TBI in war zones, including specialized rehabilitation centers, mobile clinics, and community-based programs that reach children in remote and conflict-affected areas. Additionally, psychosocial support services for both children and their families should be integrated into the rehabilitation process to address the emotional and social challenges of TBIs.

### 4.4. Limitations

As mentioned before, the major limitation of this systematic review is the analysis of biased data. However, it is important to note that it would be impossible to collect unbiased data in scenarios such as war. Additionally, the report we generated from the collected data provides a good understanding of pediatric TBIs in war zones, allowing stakeholders to develop better plans for chaotic events such as this one.

## 5. Conclusion

We found a lack of evidence-based information concerning the approach and management of children with TBIs in conflict zones, primarily due to the absence of a clear consensus on their characteristics. Our findings reveal that head injuries were the most frequently identified type of injury, often caused by blasts. The majority of affected children were reported to be from Afghanistan, with both male and female children impacted, and their average age falling between 8 and 10 years. Mortality rates varied significantly from 4 to 47%, depending on the conflict, indicating substantial diversity. In light of these findings, it is evident that as the world seeks pathways to global peace, there is an urgent need to conduct research, provide training, and deploy specialists to these regions if governments are genuinely committed to enhancing outcomes for this vulnerable population. These conclusions should serve as a catalyst for potential recommendations aimed at medical professionals, legislators, and stakeholders, urging them to ensure timely treatment and rehabilitation services for pediatric patients based on this compelling evidence.

## Data availability statement

The original contributions presented in the study are included in the article/supplementary material, further inquiries can be directed to the corresponding author.

## Author contributions

AA: Data curation, Formal analysis, Investigation, Methodology, Resources, Visualization, Writing—original draft, Writing—review and editing. KR: Formal analysis, Validation, Visualization, Writing—review and editing. AT: Conceptualization, Data curation, Formal analysis, Investigation, Methodology, Project administration, Resources, Validation, Visualization, Writing—original draft, Writing—review and editing.
